# Cockayne Syndrome Misdiagnosed as Cerebral Palsy 

**Published:** 2018

**Authors:** Amiereza VAFAEE, Taghi BAGHDADI, Sara NOROUZZADEH

**Affiliations:** 1 Department of Orthopedics, Tehran University of Medical Sciences. Tehran, Iran.

**Keywords:** Cockayne syndrome, Cerebral palsy, Prenatal diagnosis

## Abstract

A 7-yr-old patient was referred to pediatric orthopedic clinic of Imam hospital (2016) with the diagnosis of cerebral palsy (CP). His parents were concerned about some inconsistency of his disease progression. After initial evaluations, the diagnosis of CP was incorrect. The true diagnosis was suspected and confirmed with molecular genetic analysis. A rare autosomal recessive disorder -Cockayne syndrome- was diagnosed. Although untreatable, it can be prevented by appropriate prenatal diagnostic tests for their future children.

## Introduction

Cerebral palsy (CP) is a relatively common disorder with clinical presentation that predominantly becomes obvious after the first year of life (1, 2). The combination of spasticity, developmental delay, and positive birth history are the mainstays of the diagnosis. Of these, none is specific for the diagnosis and any doubt arising from clinical or history should be carefully noticed and appropriate measures are warranted.

In fact, some neurometabolic and hereditary disorders share the similar clinical characteristics with CP and only careful physical examination and detailed birth as well as developmental history could make differential diagnosis possible (3).

Here we represent a 7-yr-old boy referred to our clinic. He had been diagnosed with CP.

## Case presentation

This study was approved by the Ethics Committee of Orthopedic Surgery Department, Imam Khomeini Hospital, Tehran, Iran and a written consent was signed by the parents.

A 7-yr-old boy, the only child of otherwise healthy parents was referred the Pediatric Orthopedic Clinic, Imam Khomeini Hospital, Tehran, Iran on July 2018 with the diagnosis of CP. The reason for referral was the parents’ concern about the increasing severity of disease despite regular occupational therapy.

**Figure 1:**
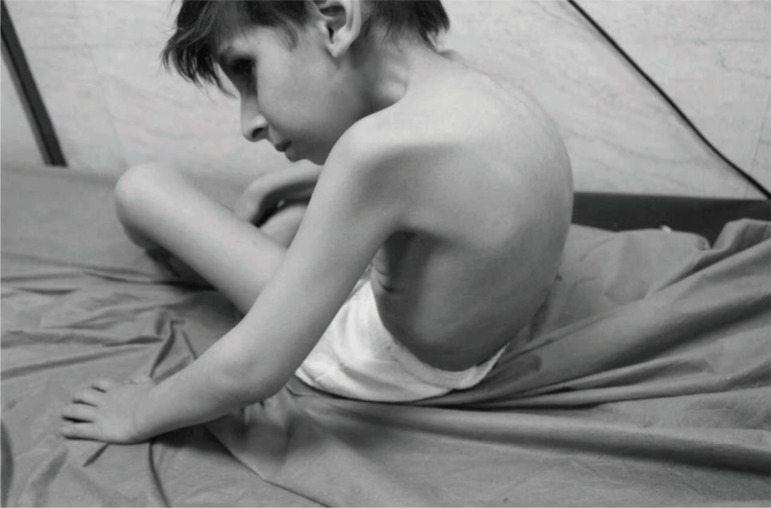
Unstable sitting balance at the age of 7 years

On physical examination, the patient was developmentally delayed, unable to walk or stand, with obvious cognitional and gross and fine motor retardation. Flexion contractures were noted in elbows, wrists, knees, and hips. There was bilateral equinovarus deformity of feet and increased popliteal angle. Plantar reflexes showed extension response and DTRs were exaggerated. Spastic response of muscles was recorded after continuous stretching. Sitting balance was extremely unstable (Figure 1).

**Figure 2:**
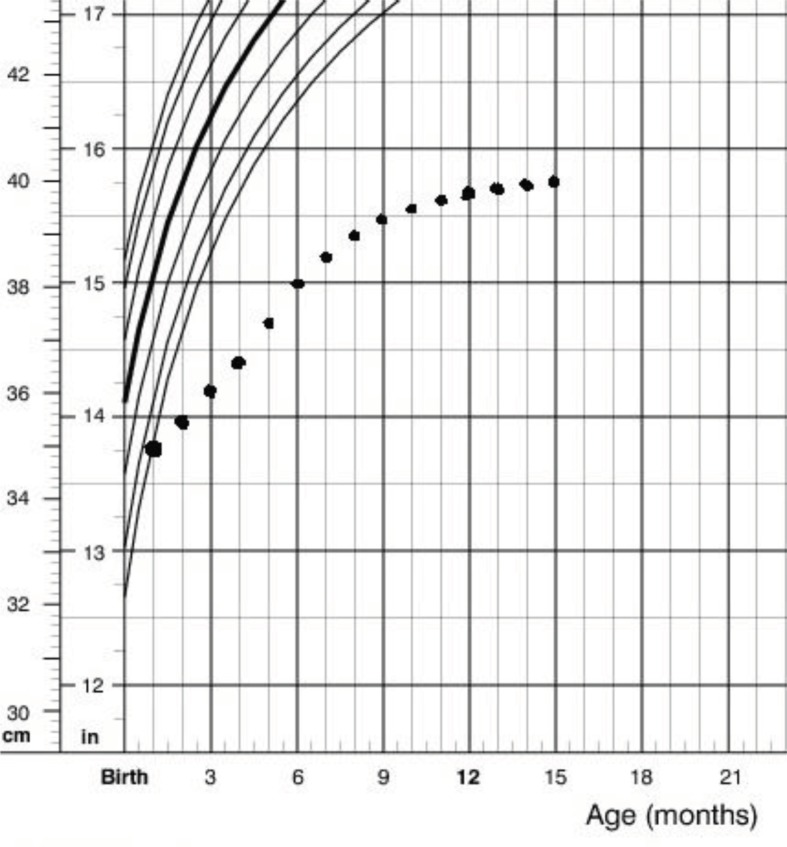
Head circumference of the patient during the first 15 months

**Figure 3:**
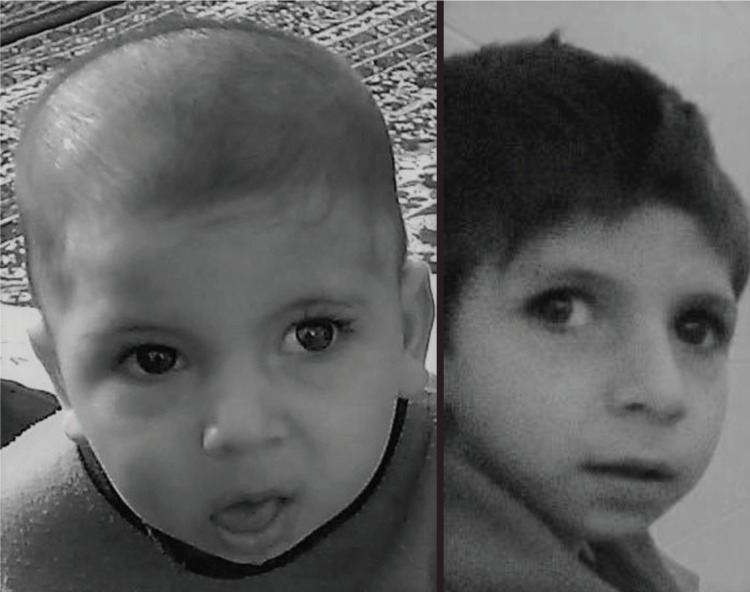
Patient face at 9 months and 7 yr old

The patient was the result of a consanguine marriage and normal pregnancy. Birth weight was 2950 gr and head circumference and height were 35 and 47, respectively. The few first months of his life showed normal weight gaining and development. He was able to hold his head in 5 months and roll over at 7 months age. The first time the parents had been told about the possibility of an abnormality was in a routine screening at 5 months age. The pediatrician noticed a decreased head circumference growth. Further investigation showed the head circumference reached a plateau (40 cm) in its growth around 12 months age (Figure 2). His general and developmental condition seemed to experience a sudden pause with progressive delay in growth and development since then. He lost his ability to rolling over and never gained any gross motor milestones. His face became expressionless and his eyes started to sink into the orbits (Figure 3). Other findings were: apparent cachectic dwarfism, microcephaly, loss of facial adipose tissue, pigmented retinopathy, thoracolumbar kyphosis, multiple joint contractures, senile appearance, photosensitivity, and thin and dry hair.

Although physical examination had a lot of similarity to a patient with CP, the history was inconsistent with the diagnosis of CP in its almost all aspects. This made us reevaluate the diagnosis. After a thorough history taking, some clues were added to our knowledge which was critical to the correct diagnosis. These include rapid regression of all motor functions, regression of language and fine motor functions and facial changes which are not compatible with CP.

At 7-yr-old age, he was in a cachectic dwarfism condition. The progeroid appearance narrowed our differential diagnosis. 

Our first diagnosis based on clinical findings and progression of the disease was Cockayne syndrome. The diagnosis was later confirmed by molecular analysis for Cockayne syndrome. The patient was homozygous for ECCR6 gene (genotype: c.2551 T>A /p.W851R- c.2551 T>A /p.W851R). The parents were also heterozygous for the same gene. This was also true for the patient’s only sister. 

## Discussion

First described by Dr. Edward Alfred Cockayne (1880-1956) in 1936, called Cockayne syndrome (CS) was initially characterized by dwarfism, retinal atrophy, and deafness (4). Through the years, many additional clinical, laboratory and genetic features are added to the disease spectrum. The fibroblasts of CS patients had an increased sensitivity to UV irradiation. The hypersensitivity was absent when these cells were exposed to X-ray radiation (5). These cell responses were also different from what is seen in patients with xeroderma-pigmentosum (6). Cells from patients with Cockayne syndrome failed to recover RNA synthesis after UV irradiation. The ERCC8 gene mutations were introduced as the molecular genetic basis of this syndrome (7). ERCC (Excision repair cross-complementing) are a group of proteins involved in DNA repair. There are different genes responsible for synthesis of these proteins. The so-called ERCC8 was the first with correlation to CS. About 80% of the CS patients have mutation in ERCC6 gene-another member of the ERCC gene family (8).

CS has been clinically classified into 4 groups (9). CSI also known as the classic CS, manifest in the first years of life. The prodromal signs and symptoms include gradual deterioration of neurodevelopmental functions, growth failure, followed by visual and hearing abnormalities, skin photosensitivity, characteristic facial changes, dental caries and eventually death in the first and second decades of life (9). CSII which is a severe form of the disease appears at birth with severe neurological findings, extensive involvement of the brain including white matter abnormalities, calcifications and atrophy. The mean age of death is 7 yr old (9). CSIII is a milder form with patients capable of living into adulthood (10). Finally, xeroderma pigmentosum-Cockayne syndrome (XP-CS) was considered with clinical characteristic of both disorders.

Another classification based on genetic abnormality classified CS into 3 groups: CSA caused by defective ECCR8 gene and CSB with ERCC6 gene abnormalities. CSC is an intermediate form combining the genetic features of XP and CS.

Whatever classification used, the basic defect in CS is the inability of cells to repair the UV induced damages to their DNA (11). Accumulation of defective DNA eventually causes cell death. Interestingly the rate of skin malignant changes does not seem to increase in CS patients (9).

Table 1 represents the main organ systems affected by Cockayne syndrome and their presence in our patient.

**Table 1:** Cockayne syndrome signs and symptoms

**Organ system **	**Abnormality**	**Presence in the patient**
CNS	Developmental/Cognitive retardation	**+**
	Inability to walk	**+**
	Decreased/increased muscle tone	**+**
	Increased reflexes	**+**
	Hearing loss	**+**
	Cranial nerves dysfunction	**-**
	Muscle weakness	**+**
	Seizures	**-**
EYES	Cataract	**-**
	Retinopathy	**+**
	Strabismus	**-**
	Photophobia	**+**
FACE	Dental caries	**+**
	Deep sunken eyes	**+**
	Fat atrophy	**+**
MUSCULOSKELETAL	Kyphosis	**+**
	Joints contractures	**+**
	Limb atrophy	**+**
	Cachectic dwarfism	**+**

**Clinical relevance**

CP is a common disorder with diverse clinical features. Although there is no specific test to prove the diagnosis, a thorough history, and physical examination could be used to rule out the less common but important diagnoses. In this case, the parents are offered to have prenatal screening test for next pregnancies. This would help them to prevent experiencing another emotionally and economically disastrous.

**In conclusion, **the diagnosis of CP must be made with caution. There are many metabolic or neurologic conditions which mimic the clinical presentation of CP with different clinical course and prognosis.
